# Review: The Development of Risk Factors and Cytokines in Retinal Vein Occlusion

**DOI:** 10.3389/fmed.2022.910600

**Published:** 2022-06-15

**Authors:** Yi Tang, Yan Cheng, Shuo Wang, Yongjie Wang, Pengjia Liu, Hong Wu

**Affiliations:** ^1^Eye Center of Second Hospital, Jilin University, Changchun, China; ^2^Department of Spinal Surgery, The First Hospital of Jilin University, Changchun, China; ^3^Australian Institute of Bioengineering and Nanotechnology, University of Queensland, St Lucia, QLD, Australia

**Keywords:** ischemic-CRVO, risk factors, neovascular glaucoma, VEGF, cytokines

## Abstract

Retinal vein occlusion (RVO) is the second most prevalent retinal disease. Despite this, the pathogenic mechanisms and risk factors are not entirely clear. In this article, we review recent publications on the classification, pathogenesis, risk factors, ischemic changes, cytokines, and vital complications of RVO. Risk factors and cytokines are important for exploring the mechanisms and new treatment targets. Furthermore, risk factors are interrelated, making RVO mechanisms more complex. Cytokines act as powerful mediators of pathological conditions, such as inflammation, neovascularization, and macular edema. This review aims to summarize the updated knowledge on risk factors, cytokines of RVO and signaling in order to provide valuable insight on managing the disease.

## Introduction

Retinal vein occlusion (RVO) is the second most prevalent retinal disease after diabetic retinopathy and can lead to vascular blindness (1, 2). The pathogenesis of RVO is not thoroughly understood and it interacts with many other diseases, but cardiovascular diseases, systemic diseases, and glaucoma have been identified as crucial risk factors (3). As the risk factors are numerous and complex, effective treatment of RVO is a major challenge. There are two types of central retinal vein occlusion (CRVO): ischemic and non-ischemic. About 20% of CRVO cases are ischemic (4). The conversion from non-ischemic to ischemic CRVO is very rapid and frequently occurs in the first month. However, it's intriguing how non-ischemic RVO transforms into ischemic RVO. Neovascular glaucoma (NVG) is a severe complication of central retinal vein ischemic-CRVO (4), often associated with vascular endothelial growth factors (VEGF) leakage from blood vessels. Cytokines and chemokines induce inflammation and neovascular in RVO because of the role of risk factors and mechanisms. This phenomenon highlights the necessity of better understanding the pathogenesis of RVO. Therefore, this paper first reviews the pathogenesis and risk factors of RVO before delving into ischemic CRVO, NVG, and cytokines/chemokines.

## Classification

Depending on the anatomical site of occurrence, RVO has two classifications: central retinal vein occlusion (CRVO) and branch retinal vein occlusion (BRVO) (1). In addition, the clinical presentation of Hemi-retinal vein occlusion (HRVO) is an intermediary between CRVO and BRVO. CRVO occurs at lamina cribrosa (2) or behind it and can be subdivided into ischemic CRVO and non-ischemic CRVO. In comparison, BRVO is mainly found in A/V intersections (2).

Ischemic CRVO is defined as the presence of more than ten disc areas of retinal non-perfusion found on retinal fluorescein angiography (FA) with standard 55°technology or revascularization on the surface of the iris (2). On the other hand, non-ischemic CRVO is characterized by <10 disc areas of retinal capillary non-perfusion (3). However, the 10-disc areas of retinal non-perfusion cannot account for regions beyond FA55°area (3). Ultra-wide area fluorescein angiography (UWF-FA) shows a larger retina surface. This detection technique will be explained in more detail in the following sections.

## Pathogenesis

Clinical manifestations reflect the disease's pathogenesis. The characteristic signs of RVO reported in current literature are: (1) Flame-shaped bleeding or intraretinal hemorrhages; (2) Optic nerve head edema, macular edema and cotton-wool spot; (3) Patients that are aged 50 or younger have a limited disease course and better final vision than those older than 50 (1, 5). Based on these characteristics, there are three plausible explanations for the pathogenesis of CRVO:

1) Mechanical lumen narrowing of thin-walled veins: The central retinal vein and artery share a common sheath at arteriovenous crossings posterior to the lamina cribrosa. Hence, when atherosclerosis thickens the artery wall, it may compress the vein and cause mechanical stenosis occlusion of the vessel wall (2).
2) Occlusion of the lamina cribrosa: In adults aged 50 years and older, the collagen tissue of the lamina is thicker and stiffer, thus compressing the vessel passing through. Meanwhile, artery degeneration can also influence the venous wall nearby. In addition to venous stasis, the narrowed vein is thought to cause turbulent blood flow and promote thrombosis formation, leading to an occlusion (6, 7).
3) Local inflammation: Vascular stasis and exudation stimulates the secretion of inflammatory factors, causing focal phlebitis and optic nerve head swelling (ONH) in a significant number of patients (3). Decreasing inflammatory factors and VEGF could relieve the disc edema and recover damaged visual acuity (VA) (8). Ischemia and hypoxia increased the oxidative stress response of the body and stimulated the secretion of inflammatory markers as well as caspase-9 (9, 10). This explains that inflammation is involved in the development of CRVO. The details surrounding involvement of caspase-9 and VEGF in the blood-retinal barrier requires further investigation (9).

## Ischemic CRVO and Non-Ischemic CRVO

Ischemic CRVO (iCRVO) is the more severe form of CRVO. More prominent manifestations of RVO are cotton-wool patches, low VA (≤ 0.1), and relative afferent pupil defect. iCRVO can cause visual loss and even neovascularization. In the differentiation of CRVO, initial vision and visual fields are essential. The initial VA of 99% iCRVO patients is 20/200 or worse, while the figure was 22% among non-ischemic CRVO (11). The severity of visual field defect was also more significant in iCRVO patients than in non-ischemic CRVO patients, especially for central scotoma. The most common defect was peripheral inferior nasal visual field defect (11). Meanwhile, central scotoma and peripheral visual defect are more severe in iCRVO than non-ischemic CRVO (11). Traditionally, ophthalmologists diagnosed iCRVO by FA. Yet now, functional test like vision, afferent pupil defect, visual field and electroretinogram demonstrate superiority (12). Ultra-wide area fluorescein angiography (UWFA) has been widely adopted to test iCRVO (13). In the study conducted by Thomas et al. (13), they used UWFA to measure baseline ischemic index (ISI) as an indicator of CRVO grading. Ischemic index, calculated on FA, is defined as the ratio of the non-perfused retina to the total visible retina (14). The classification of CRVO as ischemic based on ISI > 35% is sensitive and specific. Thus, UWFA is more useful in prognosis. A recent study shows that the disc areas and the incidence of neovascularization are positively related. Eyes that have >30 DA of retinal non perfusion have 20% increased neovascular risk (15). Retinal oxygen saturation is a non-invasive way of diagnosing iCRVO. It's based on the principle that blood color is dependent on hemoglobin oxygen saturation, and that the ischemic retina extracts more oxygen (16).

The transition from non-ischemic to ischemic CRVO is observed in 10–33% of primary non-ischemic cases. The first month has the highest risk of non-ischemic CRVO developing into the ischemic and is dependent on the initial vision and blood flow status (17). Retinal hemorrhage is greatly associated with ischemic conversion, and the severity of ischemia is used to distinguish iCRVO and non-ischemic CRVO (18). Color Doppler imaging (CDI) helps in differentiating ischemic from non-ischemic RVO through testing the minimal central retinal venous velocity (19). The progression from non-ischemic CRVO to ischemic CRVO is not fully understood but involves visual, retinociliary, and macular damage (11).

**Figure 1:**
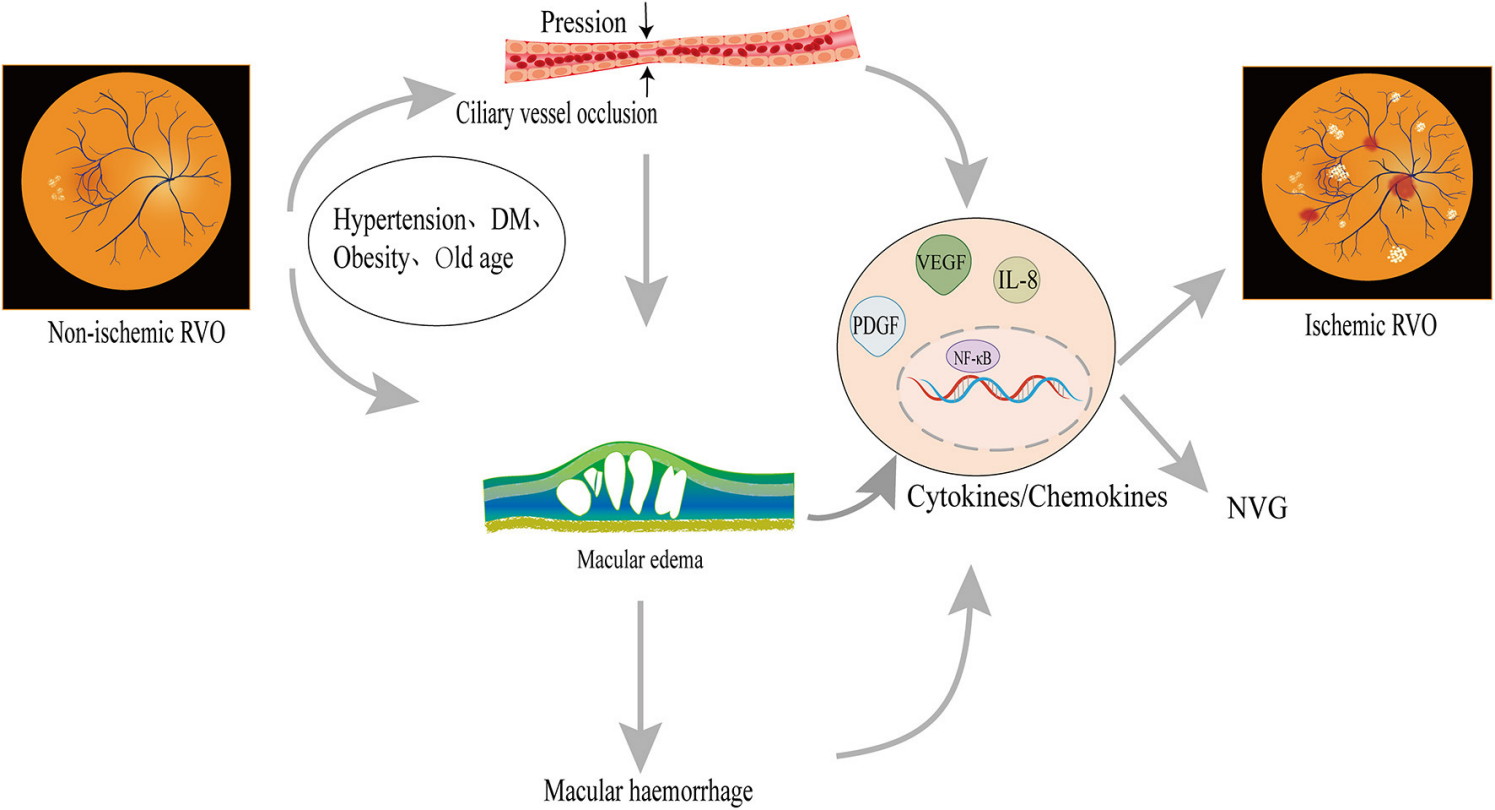
Possible mechanism of non-ischemic CRVO transforming into iCRVO: Non-ischemic CRVO along with cilioretinal artery occlusion may introduce central or peripheral visual field defects, similarly to ischemic CRVO; macular hemorrhage leads to an increase in inflammatory factors, which are involved in the occurrence of iCRVO. The occlusion of ciliary vessels induces hypoxia and VEGF production. Increased VEGF can promote angiogenesis, leading to NVG. Hypertension, advanced age, and obesity are also related to ischemic CRVO.

The transition from non-ischemic to ischemic CRVO is related to risk factors (Figure 1). Old age (20), women (>65 years old) (20), DM (21), hypertension, stroke (22), and obesity (23) are associated with a higher incidence of iCRVO. Lipocytes secrete the Adipo which monitors DM and obesity (24). Adipo plays an important role in RVO and neovascularization (25) and it might be a new treatment target. Ciliary vessels perfuse the macular region, optic nerve head and participate in the circulation of aqueous humor. Along with ciliary vessel occlusion, non-ischemic CRVO may introduce defects to the central or peripheral visual field. The non-perfusion of ciliary vessels damage macular retinal ganglion cells, which are related to the prognosis of ischemic CRVO (17, 26). When ciliary vessels are blocked, aqueous humor accumulates and intraocular tension increases, inducing glaucoma which may finally develop NVG (27). Furthermore, VEGF is an indicator to evaluate the degree of iCRVO (28). Macular edema occurs in iCRVO and non-ischemic CRVO, but ischemic injury of macular retina cells occurs in ischemic CRVO (29). Macular edema develops into macular hemorrhage, aggravating microstructure (30). The process increases VEGF, activating NF-κB and inflammatory factors (IL-8, TNF-α), thus developing into iCRVO and NVG (30, 31).

While the pathogenesis of iCRVO remains obscure, arterial hypertension, and thrombotic factors are considered the main risk factors (32). Central venous occlusion increases hydrostatic resistance, leading to blood flow stagnation and retinal ischemia injury which then increases retinal non-perfusion (RNP). There is positive feedback between VEGF and RNP (28). RNP promotes VEGF and vein occlusion while the higher VEGF becomes an important contributor to the disease by worsening retinal ischemia and thus promoting RNP. Meanwhile, retina ischemia and RNP induced permanent damage to macular retinal ganglion cells, which is the reason why VA imperfection is more severe in iCRVO. Photoreceptors are lost within the macular, leading to permanent loss of central vision and release of inflammatory material such as Interleukin 6 (IL-6), Interleukin 8 (IL-8), placenta growth factor, and VEGF. VEGF promotes new blood vessel formation in the anterior and/or posterior segments, resulting in vessel ingrowth into the vitreous cavity, ultimately, leading to secondary vitreous hemorrhage, macular edema and even NVG (33).

## Branch Retinal Vein Occlusion

**Figure 2:**
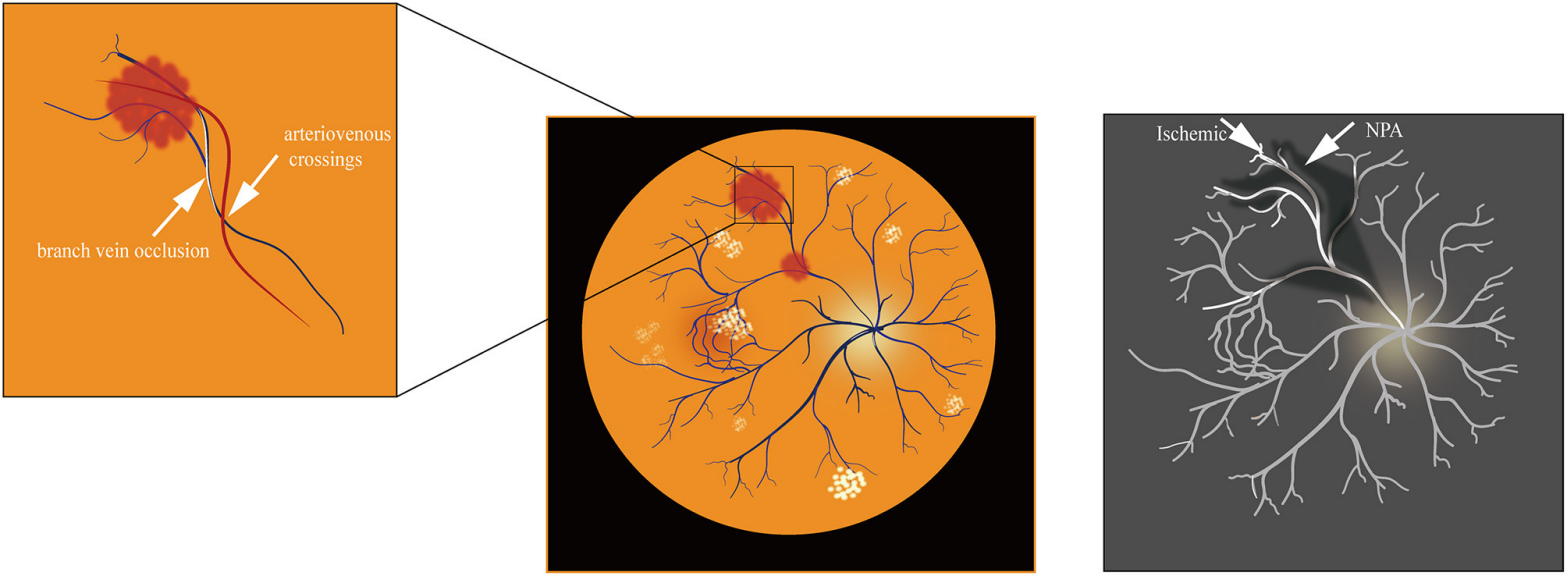
Fundus photograph and OCTA of ischemic BRVO.

BRVO typically occurs at arteriovenous crossings. At these locations, arterioles and venules share a common adventitial sheath (34). It is thought that separating arterioles and venules can restore retinal perfusion through arteriovenous crossing sheathotomy (34). Many studies have demonstrated that the underlying cause of BRVO is a mechanical narrowing of the venous lumen at intersections. Long-term hypertension results in arteriolosclerosis with thickening and hardening artery walls. This disorder then leads to further venous wall compression and narrows the lumen, causing rapid blood flow, damaging endothelial cells, increasing blood clots, and promoting vein occlusion (35, 36). BRVO is also influenced by the relative anatomical position of vessel crossovers (34, 35). Optical Coherence Tomography Angiography (OCTA) is an advanced diagnostic tool for BRVO (37). OCT imaging showed that eyes with intravenous crosses had narrower venous lumens and larger non-perfusion areas (NPA). Swept-source optical coherence tomography angiography (SS-OCTA) is sensitive to deep capillary plexuses, which helps grade the degree of macular perfusion in ischemic RVO (38–40). Ischemic retinal arteries have increased NPA of BRVO (41). At intravenous crossings, the veins anterior to the arteries were more severely compressed between the inner limiting membrane and the rigid arterial wall (Figure 2). These veins were stenotic and the NPA of the retina in BRVO was further enlarged due to venous intersections (42). Therefore, the location of arteries and veins can greatly influence BRVO pathogenesis.

## Risk Factors

### Cardiovascular

**Figure 3:**
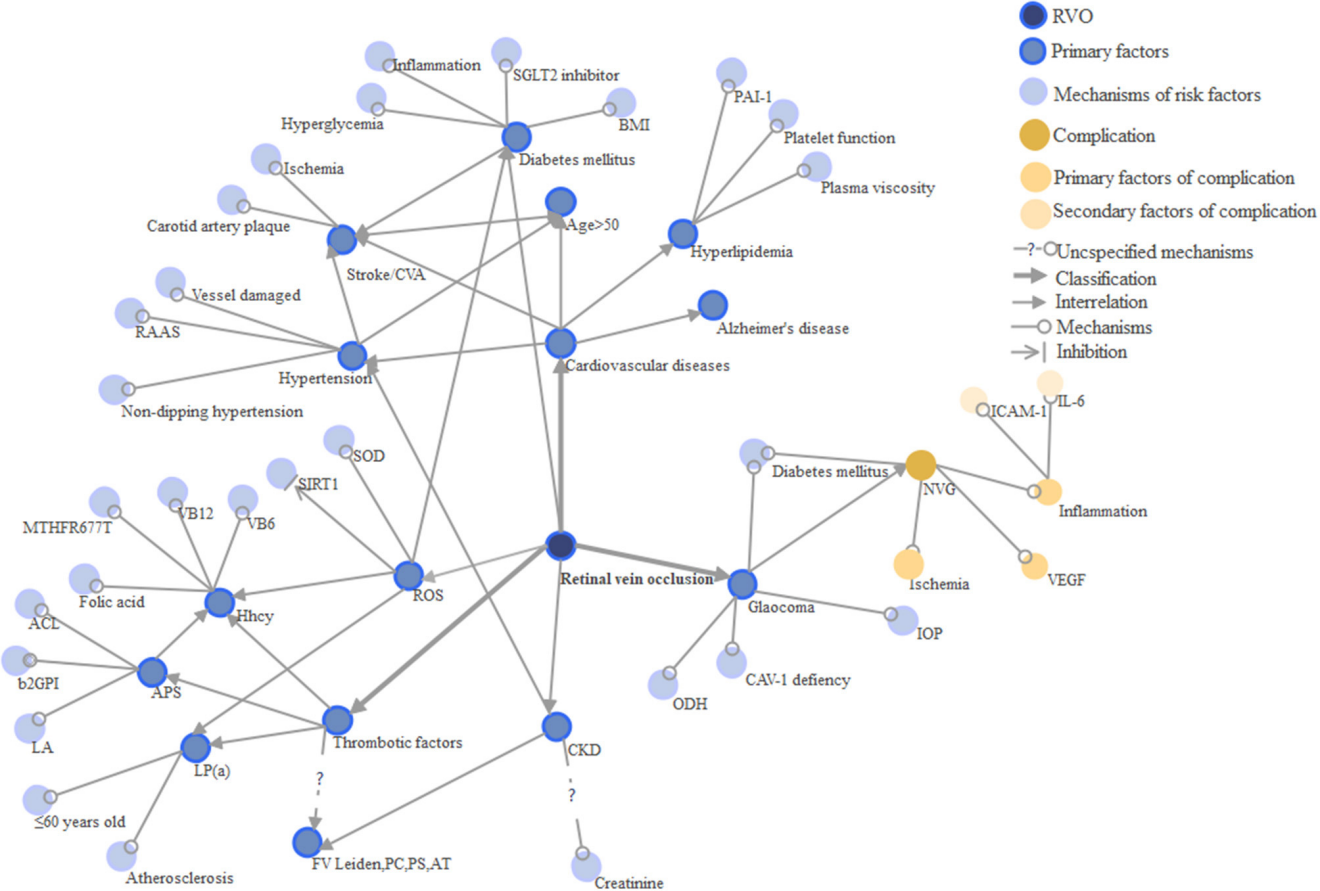
Relationships between risk factors and RVO.

Cardiovascular conditions are the most common risk factors for RVO (43) and are more likely to lead to BRVO than CRVO (44) (Table 1 and Figure 3). Hypertension, stroke, advanced age, sex, hyperlipidemia are all significant risk factors and their exact mechanisms for disease contribution are intriguing. CRVO, in particular, is thought to interact more with the cardiovascular system as it has increased cardiovascular mortality (69, 70). Because of undertreated coronary vascular and microvascular diseases, myocardial infarction (MI) and heart failure (HF) have increased in RVO (54). Age is a critical factor of RVO, as it has a positive correlation with the disease, and up to 90% of patients in the current case studies are over 50 years old (52). For females, oral contraceptives are risk factors for venous thrombosis, but prescription oral contraceptives do not increase the risk of RVO (71). Hypertension is a predominant factor of RVO among the elderly. Uncontrolled and inadequately controlled hypertension have varying influences on RVO (3, 71). Recently, ischemic stroke is also considered to be a risk factor of RVO (22, 66, 72, 73). The first 30 days after RVO development is when the ischemic or hemorrhagic stroke is most likely to occur. Thus, extra attention on preventing ischemic and hemorrhagic stroke is crucial during the first 30 days (22, 74).

**Table 1:** Characteristics of CRVO and BRVO.

**Risk factors**	**CRVO**	**BRVO**
	**Characteristics**	
Age and sex	Positively related. Mechanisms: thicker lamina cribrosa in the elderly; cooperates with cardiovascular risk factors (45).	Young patients (<50 years old) (46); it is necessary to check for thrombus factors and young patients have better prognosis.
	The prevalence of RVO is higher in women aged 55 to 84 years old (47).	
Hypertension	Uncontrolled hypertension	Prevalence in 92% (48)
	Major risk factor of RVO (20); non-dipping hypertension is the main one associated with RVO (48); patients need dynamic monitoring.	
Diabetes mellitus	53% of end-organ damage from DM (49), worsened with cardiovascular risk factors; develop ischemic CRVO and NVG.	36% of end-organ damage from DM (49), worsened with cardiovascular risk factors.
	Some medications for diabetes increase the risk of RVO: SGLT2 inhibitors (50).	
Stroke and CVA	Common (51)	Less common (51)
	Hemorrhagic stroke risk increased 30 days after RVO onset (52); closely related to ischemic stroke, and carotid artery plaque (53).	
Hyperlipidemia	Common; occurs in young patients (≤ 50 years old) (54, 55); related to PAI-1 (56).	
Oxidative stress	Common; influences the status of blood; related to DM, cardiovascular diseases and inflammation; detect ROS markers: MDA, 8-OHdG, PGC-1α, and so forth (57).	
Chronic kidney disease	Higher prevalence (58)	Lower prevalence (58)
	ESRD: 1.8% (58) Kidney transplantation decreased RVO prevalence while dialysis increased the risk (58).	
Hyperhomocysteine	Most thoroughly investigated thrombosis risk factor (59), influenced by diet (48) and mutations of MTHFR677T (60)	
	Strongly related to hypertension and ROS.	
Antiphospholipid syndrome	Multiple Apl positives (61): lupus anticoagulant (LA), anticardiolipin (ACL), and anti-b2-glycoprotein I (b2GPI); check aPL levels after a minimum of 12 weeks (62). Low-dose aspirin to treat or prevent (61).	
Lipoprotein (a)	More common in younger patients (≤ 60 years) (63), related to age, and family history of thrombosis (<45 years) (64).	
	Test: carotid ultrasound to check for carotid artery plaque and blood lipids.	
FV Leiden, PC, PS, AT	FV Leiden (65)	Deficiency of PC, PS and AT (45)
	Controversy: Studies are contradictory	
Glaucoma	Angle-closure glaucoma is positively related to CRVO (66, 67); the prevalence of PACG was 1.72% in CRVO (67).	the prevalence of PACG was 1.72% in BRVO (67, 68).
	Primary glaucoma developed RVO after 1 to 8 years (44); high IOP (66);	
	Treatment: Anti-VEGF and anti-inflammation.	

Retinal microangiopathy (stenosis of arterioles, arteriovenous incisions, and widening of venules) is associated with lacunar stroke (53) and can cause lesions in the small blood vessels of the brain. The prevalence of cerebral small vessel disease in patients with RVO is 54%, and is seen more in the elderly (≥60 years old) (75). A new study (76) reports the relationship between Alzheimer's disease and RVO. The data (76) showed that the RVO group had increased risk of subsequent all-cause dementia, Alzheimer's, and vascular dementia after adjusting for all confounding variables. Active treatment of RVO improves life quality of RVO patients. Hyperlipidemia is a risk factor for both CRVO and BRVO, and hyperlipidemia occurs more often in the young (≤ 50 years). The percentage of cases with any form of RVO attributed to hyperlipidemia was 20.1% (77). Unhealthy smoking habits cause cardiovascular disease and RVO through systematic inflammation (78). The Gutenberg RVO study (54) evaluated the relevance of multiple risk factors in patients with RVO: the most frequent combination of risk factors were hypertension with dyslipidemia and hyperhomocysteinemia with high levels of factor VIII; the risk of RVO increased by 70% with additional cardiovascular risk factors and by 40% with other types of risk factors. Hence, further studies in cardiovascular-related RVO will be meaningful both in treatment and prognosis.

#### Age and Sex

Age is an independent risk factor of RVO. Different risk factors have varying pathogenic effects on the young and the old (45). In a meta-analysis, by analyzing subgroups of different ages, they found higher risk of stroke in two groups: ages 50–59 and 60–69 (53). A study showed that younger patients (<50 years) had a better baseline and final acuities, a lower incidence of cystoid macular edema, and required fewer intravitreal injections (13). The better patient outcomes observed in younger patients can likely be attributed to less blood stasis and more active lifestyle. On the other hand, general aging and wear of organs would also lead to worse outcomes in the elderly. Increased thickness and hardness of the lamina cribrosa (where the retinal vein and arterial vein are very close together) and other cardiovascular risk factors in the elderly may additionally lead to increased risk of RVO (45). It is clear that CRVO is positively correlated with age, and it is necessary to check for thrombus factors in young patients with BRVO (46). For women, the prevalence of RVO is higher from 55 to 84 years of age (47). This finding may be related to menopause andunfavorable lipid profiles (79–81). On the other hand, in men, RVO occurs more frequently amongst those aged 30 to 54 years of age and elder than 85 years (47).

#### Hypertension

The ARIC and CHS study identified hypertension and related hypertensive retinal arteriolar changes (such as arteriovenous notch) as the major risk factors for RVO (20). Hypertension has a higher impact on BRVO than CRVO (82), and the difference is related to increased pressure at the intersection of arteries and veins. Hypertension causes RVO through pro-inflammatory mechanisms of the renin-angiotensin-aldosterone system (83). Small arteries are also damaged, leading to arteriolosclerosis and the compression of venules. This generates turbulence which causes venous blood flow stasis. In addition, the blood vessel walls damaged by hypertension changes the hematocrit, increasing the blood viscosity and thus RVO occurrence (84). Rao et al. (48) studied the relationship between non-dipping hypertension and RVO. They found that patients with RVO had almost a two-fold higher prevalence of non-dipping patterns (48). More studies are needed to further support the relationship. Uncontrolled blood pressure may be hypertension if it is normal during the day but the systolic blood pressure elevates at night. Ninety two percentage of RVO patients with hypertension have non-dipping hypertension (48). These studies on hypertension suggest that dynamic monitoring and lowering blood pressure may lower the risk of RVO.

#### Stroke and CVA

Stroke and CVA (cerebrovascular accident) are risk factors for RVO. BRVO patients are often observed to have CVA, and CVA is statistically significant in CRVO (52). Stroke is a common risk factor for CRVO (51) and the probability of stroke in RVO patients increased by 45% (73). Furthermore, hemorrhagic stroke risk increased 30 days after RVO onset (74). Hypertension retinopathy, diabetes mellitus, chronic kidney diseases are risk factors for both RVO and stroke. Therefore, it's convoluted to diagnose the pathogenesis of RVO caused by stroke. Results of RVO and stroke-related trials have been inconsistent (22, 85). One study reported that RVO was related to ischemic stroke and more frequent in younger patients (<50 years old) (22). While a study from Taiwan found that stroke patients aged 60–69 were 2.34 times more likely to develop RVO (53, 85).

Ischemia is a complex process in pathology and is regulated at the transcriptional, post-transcriptional, epigenetic, translational, or even post-translational levels (86). Circular RNAs (circRNAs) upregulate cGLIS3 in ischemic stroke, which may affect retinal neuronal function and retinal neurodegenerative processes during RVO (87). Ischemic stroke is more similar to RVO in terms of underlying mechanisms. The central nervous system (CNS) is extremely sensitive to vascular dysfunction and hypoxia as well as ischemia decreased endothelial barrier function. Dysregulation of the barrier has been implicated in stroke, Alzheimer's, RVO, and diabetic macular edema (9). CNS ischemia activates caspase-9 which promotes vascular endothelial dysfunction (9). The possible mechanisms of stroke-causing RVO could be:

1) Firstly, because retinal blood vessels are similar to cerebrovascular anatomy, physiology, and embryological features, the retina continues the diencephalon. Long-term damage to the retinal microvascular system can directly lead to cerebrovascular disease, characterized by lacunar infarct and white matter lesions (88, 89). Microvascular pathology (arteriolar stenosis, arteriovenous incisions, and widening venules) is also related to lacunar strokes.
2) Secondly, as the mechanistic reason for thrombus formation in RVO is similar to ischemic stroke, RVO is more closely associated with ischemic RVO than hemorrhagic stroke (22). Many thrombotic factors are also risk factors for stroke (53) as they are prompted to form the carotid artery plaque. Thus, the carotid artery plaque may be another source of ischemic RVO.

#### Hyperlipidemia

Hyperlipidemia is a common risk factor, especially in younger patients (≤ 50 years old) (54, 55). The prevalence of hyperlipidemia is about 20.1% (74). Changes in platelet function, clotting enhancement, and plasma viscosity may be associated with hyperlipidemia and RVO. In patients with hyperlipidemia, the activity of plasminogen activator inhibitor type 1 (PAI-1) is enhanced (56). PAI-1 is also an independent risk factor for RVO. Further research shows that the genotype of PAI-1 4G is related to RVO (56). This provides a new direction for the treatment of thrombotic RVO.

### Systematic Disease

#### Oxidative Stress

Oxidative stress is closely related to a variety of diseases such as hypertension and diabetes. Hence, its quantitative indicators lack specificity and are difficult to apply. Currently, it is used for systemic evaluation as well as evaluation of RVO severity (Figure 3).

Vascular oxidative stress is a prethrombotic state, and it triggers vascular inflammation and thrombosis (10). When venous occlusion leads to retinal occlusion and hypoxia, local oxidative stress and RVO occurs (51). Oxidative stress participates in the production of RVO by altering the state of the blood, thus checking for oxidative stress markers is an essential predictor. Reactive oxygen species (ROS) change the fluidity of red blood cell membranes, while the peroxidation of polyunsaturated fatty acids by ROS leads to the production of malondialdehyde, which then increases membrane rigidity of red blood cells (90). As the fluidity of RBC changes, its viscosity increases when passing through smaller retinal vessels, hence increasing RVO occurrence (90). The development of diabetic RVO can also be caused by oxidative stress due to increased inner membrane viscosity, the deformability of DM red blood cells is reduced (90). Superoxide anions are a major risk factor in cardiovascular conditions such as hypertension and hyperlipidemia. It is involved in basic life processes such as vascular regulation, signal transduction, and apoptosis. Homocysteine can also be oxidized to produce oxygen free radicals which damage vascular endothelial cells (48), thereby forming a thrombus. Superoxide dismutase (SOD) has the effect of anti-thrombomodulin methionine oxidation, promoting protein C activation and protecting mouse arterial and venous thrombosis (91). SOD, nerve growth factor Neurotrophin-3 (NFT3), dermal protein, and SRP14 are serum autoantibodies related to RVO (92). Through *in vitro* studies, the prethrombotic effects associated with SOD were observed to increase platelet activation, tissue factor activity, and anticoagulation disorders resulting in thrombus (91). SOD1 changes vascular tone and increases vascular permeability and vascular inflammation. Increased SOD1 can also cause acute vascular damage, leading to atherosclerosis (93), which may relate to RVO. However, SOD1 deficiency was shown to partially inhibit the activation of thrombomodulin-dependent (TM-dependent) protein C and form the thrombus. An experiment done by Weiler et al. (94) demonstrated that the lack of TM increases carotid artery thrombosis and the formation of carotid artery plaque, ultimately leading to RVO. Markers of oxidative stress in plasma such as Malondialdehyde (MDA), 8-hydroxy-2′-deoxyguanosine (8-OHdG), proliferator-activated receptor-gamma coactivator-1α (PGC-1α), and so forth are elevated in RVO patients, while the expression of superoxidase SOD is reduced (57). Hence, it is necessary to check for these markers and SOD through laboratory blood examinations. Patients with hypertension, hyperlipidemia, diabetes and Hhcy should also be treated for the prevention of oxidative stress.

#### Diabetes Mellitus

Diabetes mellitus (DM) is a critical reason for vision degradation and is the primary cause of RVO (95). Sustained high levels of glucose can lead to excessive accumulation of advanced glycosylation end products, which alter the function of the extracellular matrix, basement membrane, and vascular wall structure. These alterations result in abnormal blood vessels, including local or global arteriolosclerosis and retinal stenosis (96). Changes in end-stage diabetes mellitus might be critical to BRVO (8). Meanwhile, in DM patients who have inflammatory reactions (95), chronic diseases increase IL-6, IL-8, monocyte chemoattractant protein 1, and VEGF. Furthermore, these factors are involved in the formation of NVG, although the mechanism is not fully understood. RVO-DM patients face additional worries in terms of treatment. A recent study (50) showed that sodium-glucose cotransporter 2 (SGLT2) inhibitors increased the risk of RVO, as they altered the state of the blood. Adipo, adjusted obesity and DM, also increases in iCRVO (25). A study proved that the relationship between body mass index (BMI) and RVO depends on the severity of DM (97). Adipo might be a reason for explaining the relationship between BMI and RVO.

#### Chronic Kidney Disease

Chronic kidney disease (CKD) is an independent risk factor of RVO (98), despite having the exact pathogenesis as hypertension. After adjusting for confounders, the incidence of RVO was statistically significant in patients with end-stage renal disease (ESRD) in comparison with controls (76, 99). The prevalence of RVO in ESRD is 1.8%, and CRVO has a higher prevalence too (58). The hypothesized pathogenesis is that the retinal and glomerular filtration barriers have homologous developmental pathways and similar structural characteristics. Thus, retinal and renal circulation also have similar anatomical and pathophysiological features (34, 83). The hypercoagulable state of ERSD is associated with protein C, protein S, and Hhcy. In contrast, the occurrence of RVO may be related to the deficiency of protein C, protein S, and FV Leiden factor (45). In ESRD, the secretion of C-reaction protein, TNF-α, and IL-6 increased. Plasma tissue factor levels and fibrinogen levels were also elevated, resulting in renal and retinal coagulation and inflammation (99). CKD is also related to sclerosis of coronary arteries, leading to stasis of downstream veins and RVO. Therefore, ESRD-RVO patients should be cautious in dialysis, which changes the state of vessels (100). In the Beaver Dam Eye Study, higher creatinine was found to be a risk factor of RVO (98), while in the Blue-mountain Eye Study, the creatinine was not (101). However, patients with higher creatinine levels and renal dysfunction were indeed more likely to have RVO (102).

### Thrombotic Factors

Many thrombotic risk factors such as hyperhomocysteinemia, MTHFR gene mutation, APL, and Lp (a) were shown to be independent risk factors of RVO (60) (Table 1 and Figure 3). RVO mainly occurs in patients with a family history of thrombosis or ages ≤ 60 (64). However, it remains unknown whether the FV Leiden and the absence of PC, PS, and anti-thrombase were risk factors of RVO. The prevalence of inherited thrombophilia in patients with RVO varies according to the site of the obstruction and geographical setting (103). Yet, the mechanism of thrombosis risk factors is still not fully understood. Generally, they are linked to the process of thrombosis: blockage of blood vessels and thus triggering pathological changes in the retina. Some suggest that the investigation of hereditary thrombosis should only be considered for patients <50 years old or those without cardiovascular risk factors (104).

#### Hyperhomocysteine

Hyperhomocysteine (Hhcy) is the most thoroughly investigated thrombosis risk factor for RVO (45, 59) and it also happens to be related to other risk factors. A mild to moderate increase in serum homocysteine levels is an independent risk factor for atherosclerosis, peripheral vascular disease, and cardiovascular diseases (59). Peripheral vascular disease may then give rise to RVO. Hhcy is the cause of atherosclerosis and thromboembolism but there are no consistent results in RVO-atherosclerosis research (60, 105). Hhcy usually damages the arterial endothelium, causing aggregation of platelets, and lipids followed by thrombus formation. Furthermore, Hhcy produces free radicals because of oxidative stress, which also damages the endothelium and promotes the formation of thrombus. As a result, the thrombus causes RVO. Hhcy is a complication of hypertension, another crucial risk factor of RVO, thus it's necessary for Hhcy-RVO patients to detect hypertension for treatment. Special attention should be paid to the influence of diet on Hhcy. Hhcy can be absorbed from food, which may affect the validity of results investigating Hhcy as a risk factor for RVO (48). And Vitamins B12, Vitamins B6, and folic acid consume the serum Hhcy (59). The serum concentration of Vitamins B12, Vitamins B6, and folic may be related to the prevalence of RVO. Mutation of the 5,1-methylenetetrahydrofolate reductase (MTHFR677T) gene can affect Hhcy, which is another independent risk factor of RVO (60). The mutation rate of MTHFR677T varies from country to country (106). 5-methyltetrahydrofolate is involved in the remethylation of homocysteine to methionine. The activation of normal MTHFR can prevent the rise of Hhcy, which is also a risk factor for venous thrombosis and arterial diseases. Studies have shown that in young patients without cardiovascular risk factors, screening for MTHFR polymorphism can have diagnostic significance (48).

#### Antiphospholipid Syndrome

Antiphospholipid syndrome (APS) is an acquired autoimmune disease with thrombosis-related characteristics due to mistakenly created antiphospholipid (APL) antibodies. APS is related to ocular ischemia and retinal vascular occlusion caused by thrombosis of arterioles or venules. The mechanism of APS thrombosis is still unclear. The current view (46) is that antiphospholipid antibodies bind to β-2-glycoprotein-1, inducing the up-regulation of adhesion molecules, cytokines, and prostacyclin metabolism; oxidative LDL then damages blood vessels and binds to antiphospholipid antibodies. APL may interfere with prothrombin, factor X, protein C, protein S, plasminogen, and other proteins involved in the coagulation cascade process. This then affects the balance between coagulation factors and anticoagulation factors, hindering fibrinolysis (107). Pek- Ang et al. (108) first discovered the connection between APL and RVO which has recently been supported by more and more studies. A study showed that the prevalence of RVO in APS patients was higher than that of the control group, and the prevalence of APA in RVO patients was significantly higher too (62). There are three APLs: lupus anticoagulant (LA), anticardiolipin (ACL), and anti-b2-glycoprotein I (b2GPI). Patients with primary APS ocular vascular occlusion had high titers of IgG ACL antibody (109). Whereas, patients with multiple positives have a significantly increased likelihood of thromboembolic events (110). In the experiment conducted by Hernández et al. (61) patients with RVO-APS showed high-risk APL profiles, with a significant increase in LA and triple positive APL. Serum vitamin B12 levels of RVO-APS patients were lower than that of RVO patients without APS (61). The mechanism is unclear but may be related to Hhcy. APL and high homocysteine may be associated with an increased risk of venous thrombosis and arterial vascular diseases (64). Thus, RVO is proposed to be related to atherosclerosis but further investigation is required. Patients with RVO should be examined for primary APS (111) and in a more recent study by Rehak et al. (62), it was suggested that RVO patients should check aPL levels after a minimum of 12 weeks. Close attention should be paid to patients with a higher correlation of LA and multiple positives. There are fewer studies on pregnant women with APS hence, APL and retina testing should also be performed. Pregnancy is considered as a risk factor of RVO and pregnant women are susceptible to APS (47, 112) but there are also few studies on pregnant-APS women with RVO.

#### Lipoprotein (a)

Lipoprotein (a) [Lp(a)] (>300 mg/L) is an independent risk factor (64, 113) and the incidence is higher in younger patients (≤ 60 years) and those with a history of thrombosis (63). Lp (a) consists of a low beta lipoprotein core connected with apolipoprotein (a) and is an emerging vascular risk factor. Lp (a) affects hemolysis and promotes thrombosis, leading to increased vascular oxidative stress. Lp (a) is also an independent risk factor of cardiovascular disease. It's regarded as a threshold marker at 30 mg/dl for atherosclerosis and venous thromboembolism. Additionally, Lp (a) can enter into atherosclerotic plaque-forming foam cells resulting in apo activation and increase risk of thrombus formation (114, 115). Elevated fibrinogen promotes the migration and proliferation of smooth muscle and is related to platelet aggregation, blood viscosity, as well as directly contributing to atherosclerosis (113). Due to similarities with plasminogen structure, Lp (a) inhibits the binding of plasminogen to fibrin and endothelial cells, promoting thrombosis and atherosclerosis. It also increases oxidative stress through the production of reactive oxygen species. Furthermore, Lp (a) relates to age and family history of thrombosis. Elevated Lp (a) level is an independent risk factor in patients ≤ 60 years, patients with a history of thromboembolism, and those with family history of thromboembolism before the age of 45 (64). Lp (a) provides a lipid perspective on the prevention and treatment of RVO.

#### Factor V Leiden, Protein C, Protein S, and Antithrombin

The role of Factor V Leiden, PC, PS, antithrombin (AT) in RVO is controversial (92, 109). Kuhli-Hattenbach et al. (116) found that AT, PC, PS, and Heparin Cofactor II levels were significantly less in control groups than in patients ≤ 45 years old. On the other hand, Janssen et al. (60) found that the Anticoagulant system had nothing to do with RVO. In some studies, the difference between FV Leiden and the control group was insignificant (45, 60). While in others, FV Leiden was indeed found to be a risk factor of RVO (6). Most of these studies have limitations in that they did not adjust for confounding factors. Among RVO patients without other risk factors, the incidence of FV Leiden was significantly higher (65). The absence of PC, PS, AT is more relevant with BRVO (45), whereas FV Leiden is related to CRVO. The anticoagulant system and FV factors may be related to age, race, and family history of thrombosis. Screening for hemophilia is more valuable in RVO patients without the traditional risk factors (116). At the same time, RVO patients with hemophilia are associated with high platelet aggregation, but this only appears in a few reports (117). Whether antiplatelet therapy is effective requires more research.

### Glaucoma

Glaucoma is the leading risk factor for RVO development. Open-angle glaucoma (OAG) has a significantly correlation with CRVO (67, 118), but not with BRVO. Data shows (66, 67) that angle-closure glaucoma has a high degree of correlation with CRVO, yet the correlation with BRVO is insignificant. This may be due to the higher incidence of glaucoma in patients with optic nerve cup RVO and optic nerve RVO without nerve head swelling than in patients with arteriovenous crossover RVO. Despite the confusion about the relationship between glaucoma and RVO, Yin et al. (67) determined that glaucoma was indeed a RVO risk factor after excluding the influence of age and sex. The mechanism of glaucoma has traditionally included two theories, namely the mechanical theory and the vascular theory. Elevated intraocular pressure (IOP) (66) works on the lamina cribrosa, compressing optic nerve fibers directly. On the other hand, glaucoma compresses the retinal vein, injures the retinal vascular intima, and subsequently causes venous intimal hyperplasia. When blood runs through the lamina cribrosa, high IOP causes the retinal vein to be compressed. Unstable blood flow at the distal end of the contraction results in ischemic changes or thrombus. Anatomically, the optic nerve cup can cause the trunk of the vein to shift backwards, narrowing the capillaries. The stress due to increased IOP may be distributed within the optic nerve head through connective lamina cribrosa and cause the loss of retinal ganglion cells. The optic nerve lacks protection, which can cause venous stasis and papilledema (119, 120). Furthermore, optic disc hemorrhage (ODH) may be an important mechanism linking glaucoma and RVO. ODH is a fragmented or flame-like hemorrhage that occurs at the edge of the optic nerve or on the optic disc and is also an independent risk factor for glaucoma (105, 121). Long-term bleeding causes glaucoma which then affects the blood supply within the retina, leading to RVO. A study found that 4–5% of patients with primary glaucoma developed RVO after following up 1 to 8 years later (43). Similarly, flame-shaped hemorrhage of the optic nerve head can also be seen in RVO (3). ODH affects retinal blood flow around the papilla. When ODH occurs, retinal blood flow decreases (105) thus, ODH may cause RVO. A plausible cause of ODH is the release of vascular endothelin-1 and matrix metalloproteinases into the peripheral retinal blood vessels. When endothelin-1 and matrix metalloproteinases combined with endothelial cells, the leakage of plasma and red blood cells leads to the destruction of the blood-retinal barrier. When the blood-retinal barrier has been damaged, the innate immune system participates in RVO through release of inflammatory factors. In inflammatory retinal diseases, caveolin-1 (CAV-1) protein deficiency has been found to decrease the response of pro-inflammatory factors (IL-6, IL-8). CAV-1 increases the recruitment of immune cells, and cave-1-deficient white blood cells can enter the tight blood-retinal barrier, participating in retinal inflammation (122). CAV-1 and CAV-2 genes may be related to primary open-angle glaucoma (123). How the innate immune system is affected by RVO needs further investigation.

### Other Diseases

Instead of glaucoma, there are other diseases which might be risk factors of RVO. Retinal vasculitis may be a more relevant risk factor in young CRVO patients (124). Central Serous Chorioretinopathy (CSCR) is idiopathic and often recurs. However, the mechanism of CSCR is unclear and it's reported that CSCR patients (>40 years) increased the risk of RVO (125, 126). Regular review of CSCR patients may be the main approach to prevent the occurrence of RVO. Pseudoexfoliation syndrome (PXF) is the dominating risk factor of glaucoma and has a relationship with RVO but whether PXF is an independent risk factor of RVO requires more evidence (127). Autoimmune diseases such as psoriasis and systemic lupus erythematosus can also increase the risk of RVO (128, 129).

## Complication of ICRVO: Neovascular Glaucoma

Neovascular glaucoma (NVG) is an important complication secondary to CRVO (130) and it is related to the increase of VEGF and inflammation. The ending is eye pain, reduced vision, or even complete loss of vision. The incidence of neovascular glaucoma in iCRVO is 22–50% (131). In CRVO, the anterior segment of neovascularization is dominant, including iris, angular and neovascular glaucoma. Conversely in BRVO, neovascularization mainly occurs in the retina and optic disc (132). Nine percentage of NV developed within 9 months from BRVO onset, and 15% within 36 months (133, 134). For eyes initially categorized as non-perfused or indeterminate, 35% developed iris neovascularization/angle neovascularization (INV/ANV) (135). Usually, when INV/ANV occurs, it is treated promptly with pan-retinal photocoagulation (135). Thirty five percentage of CRVO develops neovascularization of the iris (NVI) and undergoes photocoagulation, after which 80% of eyes develop NVG (49). In BRVO laser treatment, 64% of non-perfused eyes do not have neovascularization and in eyes with neovascularization, vitreous hemorrhage was reduced from 61 to 29% (136). NVG is the neovascularization of the iris and cornea, which ultimately obstructs aqueous humor outflow and increases intraocular pressure, resulting in poor vision (27). The ischemic retina is more prone to increased vascular permeability and blood leakage. Thus, large-area optic disc non-perfusion is an important predictor of NVG. In SCORE-CRVO (137), 10 non-perfusion areas of the optic disc is the critical value for NVG. Each additional optic disc increases the risk of neovascularization by 1%. In SCORE-BRVO, 5.5 non-perfusion optic disks can trigger NVG (137). The onset time of NVG secondary to RVO is unclear and may be influenced by the severity of retinal ischemia patient status. However, the results of a retrospective study (138) indicated that the cumulative probability of NVG after RVO was 13%. The average onset time was 421 or 221 days after the final anti-VEGF injection.

One of the reasons for the formation of new blood vessels is the imbalance between VEGF and anti-angiogenic factors. Studies have shown that hypoxic retinal cells are the main source of VEGF (139). VEGF accumulated in aqueous humor promotes iris and anterior horn angiogenesis, fibrous tube infiltration, and synechiae angle closure. The outflow of aqueous humor is restricted and IOP increases, causing NVG, secondary vitreous hemorrhage, and traction retinal detachment (27, 140).

Arginyl-glycyl-aspartic acid (Arg-Gly-Asp; RGD)-binding integrins exist in the retina and are related to retinal vascular diseases. Neutralizing RGD-binding integrins weakens the penetration of the retina and choroidal vessels of VEGF (141). RGD influences retina function through the Angiopoietin/Tie (Ang/Tie) pathway, transforming growth factor-β (TGF-β), and basic fibroblast growth factor (bFGF) (141). Insulin growth factor-1 is consistent with the induction of VEGF. Inflammatory factors are also involved in the occurrence of NVG, including basic fibroblast growth factor, platelet-derived growth factor, interferon-α, IL-6, IL-8, and so on (33). Studies have found that IL-6 is involved in choroidal neovascularization (132, 137) and IL-6 levels correlate with NVG. When CRVO is combined with NVI, IL-6 in the eyes of patients increases before decreasing when the iris blood vessels recede (142). The synchronized changes in IL-6 and VEGF levels indicate that there is a causal relationship between IL-6 and VEGF (143, 144). Intercellular Adhesion Molecule-1 (ICAM-1) is a transmembrane protein related to endothelial cells and white blood cells and has predictive value for the treatment response of a variety of macular diseases. After the injection of anti-VEGF, the VEGF in the aqueous humor was significantly reduced, but the levels of ICAM-1 and IL-6 did not change. The angiogenic pathways involving ICAM-1 and IL-6 need further investigation (138). Anti-VEGF therapy can only delay the occurrence of NVG, not prevent it (17, 142). This means the simultaneous use of both anti-VEGF and anti-inflammatory factors may be more effective in treating NVG.

Age is an important risk factor that affects the development of NVG but its mechanism is unclear (33, 131). Rong et al. (131) found that age was not significant when sorting patients into two groups: those over 50 and those under. In Tam's experiment (33), it was pointed out that the degeneration of photoreceptors was due to old age. As age leads to worse vision this also helps explain the higher incidence of iCRVO among the elderly. Systemic hypertension and free radicals in aqueous humor also have a strong correlation with NVG. Although macular edema is also an important manifestation of iCRVO, it has been proved to be independent, unrelated sequelae of CRVO. DM is a risk factor for RVO and promotes NVG. DM is also closely related to carotid plaque formation and long-term hyperglycemia which leads to microvascular disorders, increasing the risk of vascular occlusion. ICRVO follows resulting in vascular leakage. Increased VEGF exudation causes neovascularization, and NVG happens secondary to iCRVO (145).

## Cytokines and Chemokines

**Figure 4:**
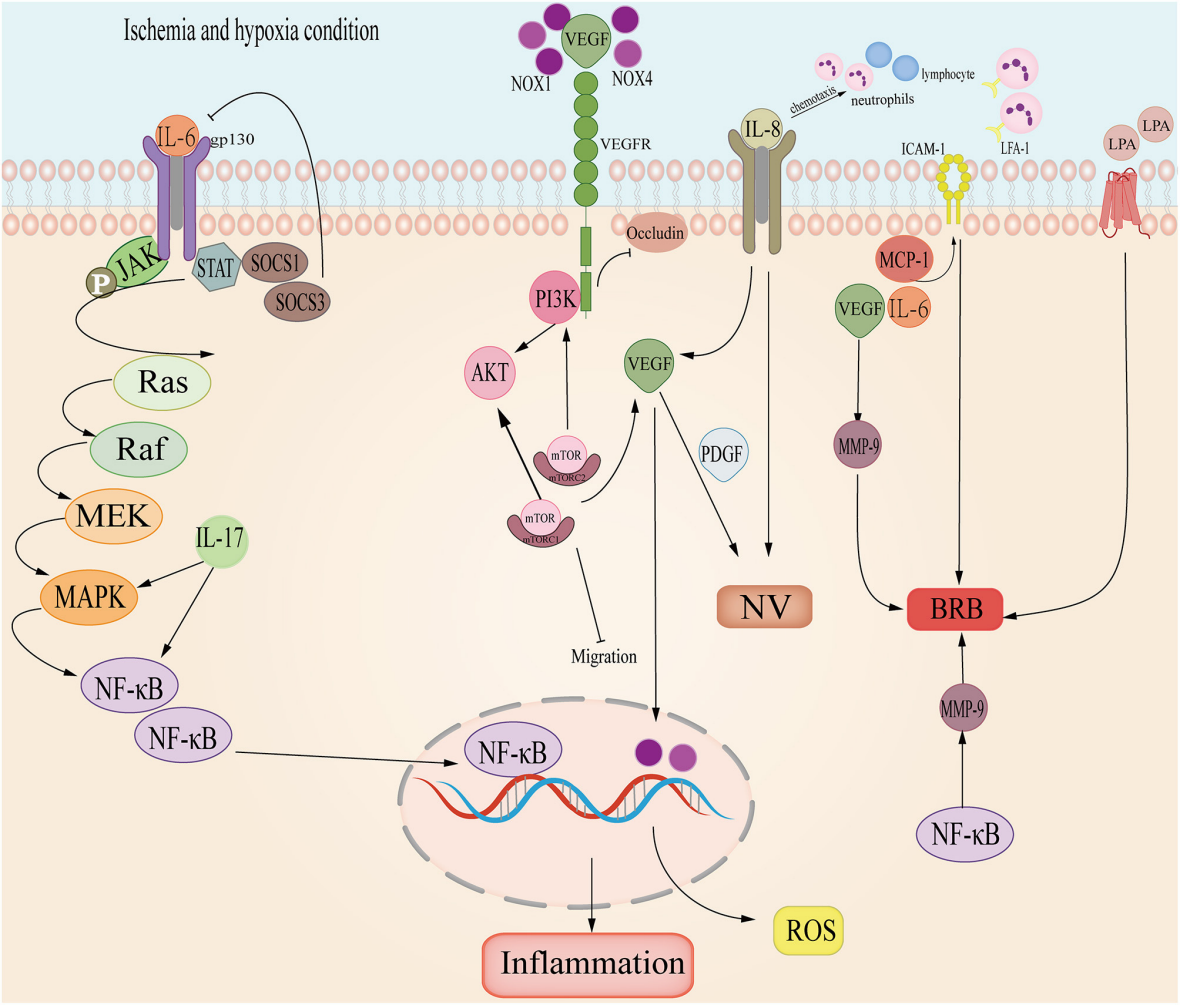
The cytokine and chemokine pathways involved in RVO. Interleukin-6 (IL-6) promotes Janus-activated kinase/MAP kinase pathway (JAK/MAPK) and induces NF-κB, causing retina inflammation; Interleukin-6 (IL-8) induces VEGF and leads to neovascular (NV); in ischemia and hypoxia, VEGF and PDGF promotes NV; NOX combines with VEGF, inducing PI3K/AKT and ROS; VEGF-VEGFR-2 inhibits occludin damaging the basal membrane of endothelial cells; Intercellular Adhesion Molecule-1 (ICAM-1) cooperates with VEGF, MCP-1 and IL-6 induces BRB; LPA- ATX may participate in RVO.

Much research has demonstrated that cytokines and chemokines in the aqueous humor and vitreous were significantly correlated with RVO, including the Interleukin family, VEGF, Matrix metalloproteinase, and LPA. We reviewed these factors in detail (Table 2 and Figure 4).

**Table 2:** Cytokines and chemokines in RVO.

**Cytokines/chemokines**	**Generation**	**Major activities**
IL-6	Ischemia, vessel injury	IL-6 causes macular edema secondary to BRVO. IL-6 combines with IL-6R and gp130, stimulating STAT3, MAPK, NF-κB and VEGF. (146–148).
IL-8	Ischemic and macular edema	IL-8 promotes neovascularization, chemotactic neutrophils and lymphocytes. IL-8 is sensitive to ischemic and macular edema is linked with IP-10, MCP-1, and VEGF. (31, 149, 150).
IL-17	Inflammation	IL-17 engages in ME and destroys BRB. IL-17 provokes ROS in hypoxia and ischemia and triggers NF-κB and MAPK signal pathways (151–153).
IL-18	Destroyed muller cells and proliferated retinal capillaries	IL-18 promotes ICAM, NO, and chemokines. The rise of IL-18 is especially pertinent in damaging muller cells and proliferation of inner layer retinal capillaries (154, 155).
VEGF	hypoxia and ischemia	VEGF-VEGFR stimulates PI3K/Akt and mTOR, and mTOR promotes the secretion of VEGF. VEGF changes the vascular permeability: occludin damaging, MMP-9 activates, and VEGF induces ICAM-1 causing leukocytes stasis. VEGF activates NOX in the endothelium and promotes ROS (156–160).
MMP	In the RVO inflammation model and vascular hyperpermeability, leukocytes secrete MMP-9.	MMP is aroused when clotting forms in RVO. MMP-9 is downstream of NF-κB and degrades the basement membrane (158).
LPA-ATX	Inflammation	LPA-ATX activates IL-6, IL-8, VEGF, MMP-9 and MCP-1. It may be linked with BBB breakdown (29, 161).
PDGF	Generated by retinal ganglion cells	PDGF-A prevents ischemic retinopathy by promoting retinal glial proliferation. PDGF-A reinforced VEGF to induce neovascularization (162–164).

### Interleukin Family

Interleukin is involved in the inflammation process of RVO. Especially, IL-1, IL-6, IL-8, IL-12, IL-15, IL-18 which are all seen to be up-regulated in RVO (31, 151, 165).

As a pro-inflammatory factor, Interleukin-6 was up-regulated in RVO, especially in CRVO but decreased in macular edema secondary to RVO (31). In the retina, IL-6 is related to ischemia and vessel injury. In BRVO, IL-6 is significantly related to NPA (166) and caused macular edema secondary to BRVO (146). During inflammation, IL-6 binds with IL-6R forming a compound which then combines with gp130 and activates the downstream STAT3 and JAK-MAPK pathways. STAT3 induces the transduction of SOCS1 and SOCS2, inhibiting the activation of IL-6 (147). Meanwhile, IL-6 stimulates NF-κB and VEGF, which are directly relevant to macular edema (148).

IL-8 is also a pro-inflammatory factor and it can promote neovascularization. It has a chemotactic effect on neutrophils and lymphocytes (149). Research shows that IL-8 is more sensitive to ischemic and macular edema (31). IL-8 is also linked with IFN-γ which induces proteins IP-10, MCP-1, and VEGF (150) in RVO aqueous humor. In view of these mechanisms, IL-8 plays an important role in BRVO and ischemic RVO, thus deserving research and clinical attention.

IL-17 is a proinflammatory cytokine, including Il-17A, IL-17B, IL-17C, IL-17D, IL-18E, IL-17F. It's proved that IL-17could trigger NF-κB and MAPK signal pathways (152). In BRVO, IL-17E engaged in ME together with MCP-1 (151). Meanwhile, IL-17A could destroy BRB and provoke ROS in hypoxic and ischemia conditions (153). The blood-brain-barrier (BBB) is anatomically similar to BRB, thus IL-17 may also play a role in damaging the BBB.

IL-18 and S100A12 might be the cause for ischemic inflammation. During inflammation, IL-18 promotes ICAM, NO, and chemokines (154). Monocytes, glia and dendritic cells all produce IL-18 (167) and in the retina, IL-18 can be secreted by muller cells. The rise of IL-18 is especially pertinent to damage by muller cells and proliferation of retinal capillaries in the inner layer of the retina (155). S100A12 plays an important role in leukocyte adhesion, migration, and chemokine and cytokine production (168, 169). S100A12 is abundant in retinal ganglion cells (167), which promotes inflammation in the posterior segment.

### VEGF

In hypoxia and ischemia, VEGF increases to form neovascularization and counter hypoxic-ischemic conditions. During RVO, VEGF is the main cytokine that induces ischemia and neovascularization (170, 171). Because of hypoxia, ischemia or blood stasis, vascular permeability increases and VEGF exudes. VEGF binds to VEGF-R stimulating PI3K/Akt which then induces a mechanistic target of rapamycin (mTOR). mTOR curbs the migration of APRE cells, and mTOR triggers VEGF and PDGF secretion (156). VEGF induces ICAM-1 to cause the stasis of leukocytes and alters vascular permeability (157). In macular edema secondary to BRVO, VEGF cooperates with ICAM-1, IL-6, and MCP-1 to impair the BRB (146, 148). In NVG secondary to RVO, VEGF-VEGFR2 suppresses occludin to damage intercellular tight junctions and activates MMP-9 to destroy BRB (158). Furthermore, VEGF is an essential exudation factor and can promote atrial adhesion hyperplasia and angiogenesis, resulting in NVG induced by high IOP (27, 140). VEGF also increases oxidative stress. NOX1 and NOX4 proteins dominate ROS generation in RVO (159). and upregulate VEGF. However, VEGF activates NOX in endothelial and ROS accumulation (160).

### Matrix Metalloproteinase

MMP promotes BRB degradation and thrombosis. It is a downstream target of NF-κB/TNF-α which degrades the basement membrane (172). In a RVO inflammation model and vascular hyperpermeability model, leukocytes secrete TNF-α and MMP-9 (158). Moreover, MMP and coagulation factor activities are aroused when clotting occurs in RVO. At the same time, elevated heparinase levels activate TLR and trigger the release of MMP-9, promoting the generation of Xa (173). Additionally, MMP-2 and MMP-7 were proved to be involved in the migration of vessel endothelial cells (174). Thus, the MMP family may play an important role in inflammation and angiogenesis of RVO.

### LPA-ATX Signal Pathway

Lysophosphatidic acid (LPA) interacts with 6 specific G protein-coupled receptors to transmit extracellular signals (172). LPA mediates inflammation, apoptosis, cell migration, angiogenesis, and secretion of cytokines and chemokines. Studies showed that the LPA signal pathway affected neovasculature in the CNS and changed the permeability of the endothelial layer, thus, breaking the BBB (173, 174). ATX is the main source of LPA, and there is an ATX-dependent and independent way to synthesize LPA (173). BRB is analogous to BBB in anatomical structure and physiological function. Experiments showed that a broken BBB caused elevated ATX levels in serum (175). LPA-ATX may mediate inflammation in RVO (29) as it activates IL-6, IL-8, VEGF, MMP-9, and MCP-1 (29). The LPA-ATX signal pathway is convoluted and involves the Hippo pathway (176). Further analysis of LPA-ATX signaling is necessary.

### Platelet-Derived Growth Factor

Platelet-derived growth factor (PDGF) is generated by retinal ganglion cells, protecting the BRB and endothelial layer (177). PDGF-A prevents ischemic retinopathy by promoting retinal glial proliferation (177). However, PDGF-A cooperated with VEGF to induce neovasculature hence it could be a new target for angiogenesis in combination with VEGF (161, 178).

## Treatment and Management

The current treatments of RVO include macular mesh laser therapy, cortisol and anti-VEGF. Anti-VEGF is the most common treatment and includes drugs such as Conbercept, Ranibizumab and Bevacizumab which generally demonstrates safety and efficacy in clinical treatment (160, 162–164). Anti-VEGF is also the standard therapy of ME secondary to RVO and can help improve choroid thickness too (179). However, the timing and frequency of anti-VEGF greatly impacts RVO prognosis (180–182). Early and long-term therapy with 6 injections of ranibizumab monthly or every 2 months has shown to be effective in treating ME secondary to RVO and BCVA (180). Post-anti-VEGF injections, the visual acuity appears peaks and subsequently decreases (183). The mechanism of RVO is complex includes VEGF and inflammation, as a consequence, anti-VEGF is not effective on all patients. Patients who do not respond to anti-VEGF therapy are recommended other therapies such as: Intravitreal injection of cortisol (184–188); anti-adiponectin improves angiogenesis *in vivo* (25); Laser photocoagulation combined with anti-VEGF intravitreal injections (184). Alternatively, another important approach is to improve risk factors such as through ambulatory blood pressure monitoring and metformin blood glucose control (189). At present, more attention should be put on the therapeutic regimen, prognosis and the dire issue of how RVO patients can improve their intraocular status (190) when they cannot continue treatment during COVID-19.

## Conclusion and Prospect

The article reviews the following: (1) the classification of RVO; (2) the transition from non-ischemic to ischemic CRVO; (3) the influence of risk factors on RVO (Table 1); (4) the mechanisms of RVO: (4.1) Ischemia and hypoxia; (4.2) inflammation in local retina; (4.3) the state of blood and vessel damage; (5) NVG secondary to RVO: Ischemia and hypoxia induce neovascular formation and inflammation in NVG through VEGF and ICAM-1; (6) cytokines and chemokines: IL-6, IL-8, IL-17, IL-18 are pro-inflammatory factors; VEGF, PDGF, MMP family, and LPA-ATX cause the BRB breakdown in RVO; (7) treatment and management.

Even with abundant research into the risk factors of RVO the exact mechanisms are still obscure, especially since these risk factors are heavily interrelated. There are still many challenges to be overcome in regards to management and treatment of the disease. It is necessary to study the cooperation of cytokines that activate both relevant risk factors and RVO itself. These cytokines play crucial roles in uncovering the disease mechanism and could prove to be promising treatment targets. For example, hypoxic-ischemia in the CNS triggers caspase-9 to damage the endothelial layer. Based on the consequences, a study (9) proved that caspase-9 also damaged the optic nerve cell in iCRVO. Further studies of other factors such as LPA, carotid plaque, irisin and Circular RNAs all show involvement in the occurrence of RVO (29, 53, 87, 191). The study of etiological factors behind RVO is also extremely important in bettering treatment, prognosis as well as prevention.

## Method of Literature Search

Two databases were searched including Pubmed and Web of Science with no year limitations. Keywords included retinal vein occlusion, ischemic, treatment, risk factors; these were then combined with cardiovascular diseases, chronic kidney disease, or thrombotic factors. Further searches were conducted combining the stated keywords with epidemiology, prevalence, incidence, cytokines and chemokines; mechanisms, pathogenesis, neovascularization, diagnose technologies, and the different therapies for the management section.

## Author Contributions

YT and HW conceived the review project. YT devised the manuscript. SW and YW directed the writing. YC, PL, and HW critically discussed the review. All the above-listed authors edited the manuscript. All authors contributed to the article and approved the submitted version.

## Funding

This work was supported by the Department of Science and Technology, Jilin Province (20190701076GH and 20200404103YY).

## Conflict of Interest

The authors declare that the research was conducted in the absence of any commercial or financial relationships that could be construed as a potential conflict of interest.

## Publisher's Note

All claims expressed in this article are solely those of the authors and do not necessarily represent those of their affiliated organizations, or those of the publisher, the editors and the reviewers. Any product that may be evaluated in this article, or claim that may be made by its manufacturer, is not guaranteed or endorsed by the publisher.
